# Videocapillaroscopy of the Oral Mucosa in Patients with Diabetic Foot: Possible Diagnostic Role of Microangiopathic Damage?

**DOI:** 10.3390/jcm9113641

**Published:** 2020-11-12

**Authors:** Giuseppe A. Scardina, Giovanni Guercio, Cesare F. Valenti, Domenico Tegolo, Pietro Messina

**Affiliations:** 1 Department of Surgical Oncological and Stomatological Disciplines, University of Palermo, 90133 Palermo, Italy; giovanni.guercio@unipa.it (G.G.); pietro.messina01@unipa.it (P.M.); 2 Department of Mathematics and Informatics, University of Palermo, 90133 Palermo, Italy; cesare.valenti@unipa.it (C.F.V.); domenico.tegolo@unipa.it (D.T.)

**Keywords:** diabetic foot, oral videocapillaroscopy

## Abstract

Introduction: Diabetic foot represents one of the most serious and expensive complications of diabetes and is subject to a high percentage of amputations that are almost always preceded by ulcers ascribable to neuropathy and/or vasculopathy. Videocapillaroscopy (VCS) can be a valuable aid in order to uncover morpho-structural anomalies in the vascular bed, both at the level of the oral mucosa and at the level of the terminal vessels of the lower limb. Materials and methods: Sixty subjects divided into 4 groups were enrolled: 15 healthy subjects; 15 patients with diabetes for more than 10 years without ulcerative foot lesions; 15 patients with neuropathic diabetic foot (clinical diagnosis, MDNS); 15 patients with ischemic diabetic foot (clinical diagnosis, ABI, lower limb doppler). A complete videocapillaroscopic mapping of the oral mucosa was carried out on each patient. The areas investigated were: labial mucosa, the retro-commissural region of the buccal mucosa, and the vestibular masticatory mucosa (II and V sextant). Results: The analysis of the morphological and densitometric characteristics of the capillaries revealed the following: a significant reduction in capillary density in neuropathic (mean ± SD 7.32 ± 2.1) and ischemic patients (mean ± SD 4.32 ± 3.2) compared to the control group of patients (both diabetic mean ± SD 12.98 ± 3.1 and healthy mean ± SD 19.04 ± 3.16) (ANOVA test and Bonferroni *t* test *p* < 0.05); a reduction in the average length of the capillaries and a significant increase in tortuosity (ANOVA test and Bonferroni *t* test *p* < 0.05). In the neuropathic patients, a recurrent capillaroscopic pattern that we defined as “sun” was found, with capillaries arranged radially around an avascular area. Conclusions: The data obtained from this preliminary study suggest a potential diagnostic role of oral capillaroscopy in the early and subclinical identification of microangiopathic damage in patients with diabetic foot.

## 1. Introduction

Diabetic foot (DF) represents one of the most serious and expensive complications of diabetes and is subject to a high percentage of amputations [1,2,3,4].

DF is a source of major suffering and financial costs for the patient and also places a considerable charge on the patient’s family and society in general. The prevalence of foot ulcers among diabetic patients ranges from 3% to 13% globally. There are many factors involved in the development of foot ulcers in patients with diabetes. The two most important risk factors are peripheral neuropathy and peripheral vascular disease. The role of microangiopathy remains controversial given the difficulty of in vivo studies of microcirculation [5,6,7,8,9,10,11]. Microangiopathy may play a significant role in the pathogenesis of tissue breakdown in the diabetic foot. However, the precise mechanisms of this process remain unclear and poorly understood [12].

Insufficient microcirculation and infection turn the chronic wound into gangrene. Trauma also plays a significant part in the development of ulceration. Diabetic foot ulcers are a common complication in patients with uncontrolled diabetes and are the most common cause of non-traumatic amputations [4,8,9,10]. Evidence in the literature suggests that the early detection and treatment of diabetic foot complications could reduce the prevalence of ulceration by 44% to 85%. Early diagnosis can reduce the burden of diabetic foot disease. Early screening of high-risk patients is important to prevent development of foot ulcers and its associated morbidity. One of the factors closely linked to diabetic foot infections could be impaired microcirculation [11].

Evaluating peripheric microcirculation in patients with diabetes can help to develop a treatment plan that can help avoid complications like amputations. 

Videocapillaroscopy (VCS) is a useful tool to investigate microcirculation “in vivo” even in patients with chronic metabolic and inflammatory diseases [13].

Oral videocapillaroscopy is a simple, reproducible, non- invasive, panoramic technique that is performed in vivo and is well-tolerated by the patient.

The alterations of microcirculation in diabetes is of a generalized nature, VCS can be a valuable aid in order to uncover parametric and non-parametric morpho-structural anomalies, in the vascular bed, both at the level of the oral mucosa and, probably, at the level of the terminal vessels of the lower limb [12,13]. Studies in the literature have shown a correlation between diabetes and oral microcirculation. The in vivo study of oral microcirculation is a decidedly simpler approach compared to the study of the vascular pattern in other peripheral areas. The main reason is linked to the lack of a stratum corneum in some areas of the oral mucosa, optimizing the visualization and therefore the evaluation of the capillary bed. It is not possible to observe the vascular pattern at the lower extremities with the videocapillaroscopic approach.

The aim of this research was to study the oral microcirculatory pattern of healthy patients, diabetic patients and diabetic patients with neuropathic and ischemic complications and subsequently evaluate any correlations between the observed oral microcirculatory pattern and the diabetic complications that can be found in the foot.

## 2. Materials and Methods

All subjects gave their informed consent. This observational cross-sectional research was conducted in accordance with the Declaration of Helsinki and the protocol was approved by the Ethics Committee of Policlinic Palermo N#02/2010.

The subjects were randomly recruited (from 2014 to 2018) at the Department of Surgical Oncological and Stomatological Disciplines.

Sixty subjects (age and sex matched) were divided into 4 groups: 15 healthy subjects (HS); 15 patients with diabetes for more than10 years without ulcerative foot lesions (WFU); 15 patients with neuropathic diabetic foot (clinical diagnosis, MDNS) (NDF); 15 patients with ischemic diabetic foot (clinical diagnosis, ABI, lower limb doppler) (IDF) (Table 1).

Healthy subjects were recruited from among the volunteers. Since the capillaroscopic survey is absolutely non-invasive, it was simple to enroll the subjects. Diabetic patients were randomly recruited within their membership group: WFU, NDF, IDF.

Neuropathic and vascular tests, biotensiometer, Systoe, transcutaneous oxygen pressure, were used to confirm the diagnoses.

There were no statistical differences in the duration of diabetes, glycemic and lipidic level, blood pressure for the enrolled subjects. Diabetic patients took insulin or oral hypoglycemic drugs.

A complete videocapillaroscopic mapping of the retro-commissural region of the buccal mucosa, vestibular masticatory mucosa (II and V sextant) and labial mucosa was carried out on each subject.

### 2.1. Inclusion Criteria

- Patients without foot ulcers with a history of diabetes of more than 10 years (WFU), coding of diagnosis type 1 or 2 was established, based on clinical/GP records (HbA1c);
- Patients with neuropathic diabetic foot (diagnosed through positive results in the Semmens-Weinstein monofilament test (10 g monofilament to determine the loss of protective sensations-Darco footfilament), absence of patellar and Achilles reflexes, presence of deformity and possible plantar ulcers) (NDF); the patients have to meet all these criteria for consider them as neuropathic;
- Patients with ischemic diabetic foot (diagnosed on the basis of clinical parameters like color and temperature and doppler examination of the vessels in the lower limbs; positive markers like Ankle-Brachial Pressure Index (ABPI) were also used) (IDF).

### 2.2. Exclusion Criteria

- Patients with oral pathologies (candidiasis, lichen planus, glossitis, periodontitis, etc., so patients had a healthy mucosa) and systemic pathologies (vitamin and mineral deficiencies, autoimmune disorders, etc.);
- Patients with previous vascular intervention e.g., femoral bypass graft/ treated vascular disease were excluded;
- Smokers, patients who had reported a previous appearance of mycosis, hypertensive patients (because of the collateral effects of their pharmacological therapy) and, in general, patients submitted to daily pharmacological treatments (except those for diabetic disease).

Each patient signed the informed consent for capillaroscopic analysis and the consent to enroll in the experimental study, in accordance with Italian laws and with declaration of Helsinki good clinical practice.

A complete capillaroscopic examination of the oral mucosa was carried out for each patient.

**Figure 1:**
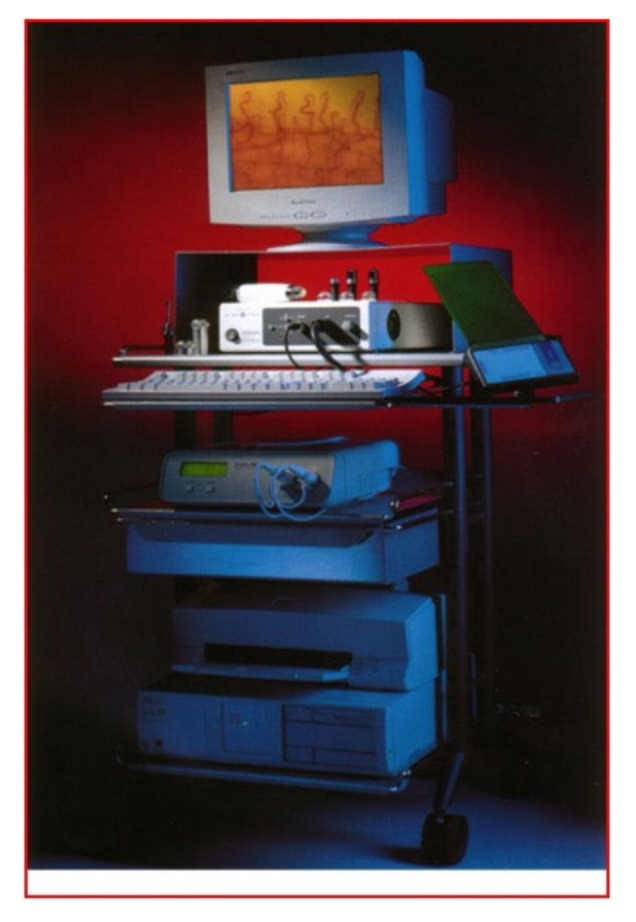
Videocap 100.

The capillaroscope used in this study was the Videocap 100 (DS MediGroup, Milan, Italy) (Figure 1). The video microscope consists of:

- A central body, containing the light source (halogen lamp), the video capture electronics, the main manual controls for adjusting brightness, contrast, color balance, etc.;
- An optical probe with a 200× terminal and 2 m long cord. The probe has a rotating focusing wheel;
- A PC with dedicated software (VideoCap software version 8.1) capable of capturing, storing and editing capillaroscopic images.
- The sites investigated for each patient were the following:
- Labial mucosa, at the level of the lower lip;
- The retro-commissural region of the buccal mucosa;
- Masticatory mucosa (adherent alveolar mucosa in the case of an edentulous patient).

We obtained two images for every site and they were all evaluated.

All the investigations were carried out by the same operator (GAS) and in the same temperature conditions (25 °C), with constant lighting and in the morning hours.

Two independent observers looked at all the pictures, performing double blind evaluations for the subgroups. To limit intra and inter examining variables, the two observers evaluated the same randomly selected images twice. The two observers were not calibrated and the differences in the observed data were not statistically significant.

**Figure 2:**
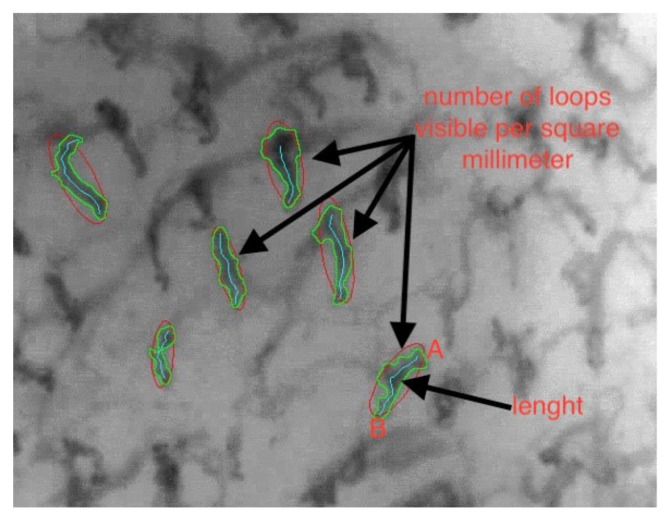
Parametric data: number of loops and length.

**Figure 3:**
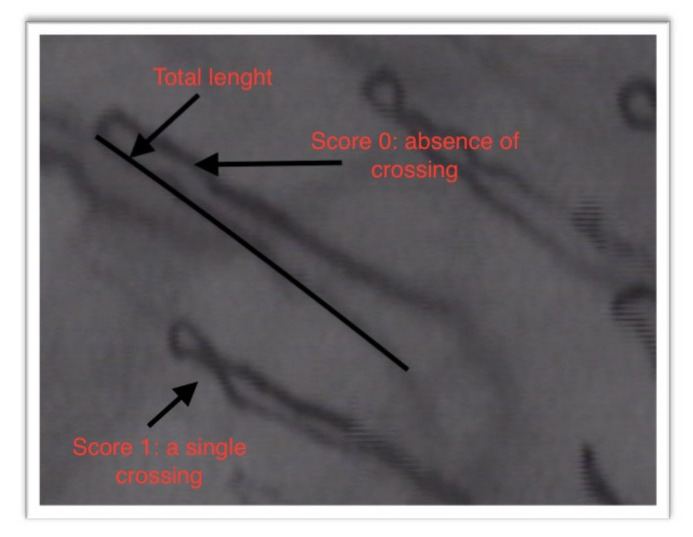
Parametric data: length; non parametric data: the tortuosity of the capillary loops.

Taken into consideration were the following parametric data:

- The density of the capillary loops (the number of loops visible per square millimeter) (Figure 2);
- Total length of the capillary loops and their size (Figure 3);
- The degree of tortuosity of the capillary loops: the evaluation of the tortuosity of the capillary loops was carried out by attributing points from 0 to 3, according to the number of crossings that they had:

Score 0: absence of crossing
Score 1: a single crossing
Score 2: more than double crossings
Score 3: distorted loops

and the following non-parametric data:

- Presence/absence of capillary loops with atypical morphology.

It is important to emphasize that the parametric data originate from software related to video capillaroscopy using a dedicated measuring instrument, each optical magnification corresponding to an exact value of metric pixels in the scanned image [13,14,15].

Data collection and statistical analysis was performed with the help of the Open Office 4.1.3 program. A statistical analysis was performed using PAST software (version 3.18 updated in August 2019, Øyvind Hammer, DAT Harper and PD Ryan). The statistical significance of the differences was checked with the ANOVA test and ANOVA post-hoc test (Bonferroni test t). The level of significance was set to *p* < 0.05. Differences with a *p*-value less than 0.05 were considered statistically significant. Parametric data, e.g., density, length, tortuosity of capillary loops was analyzed by the VCS software.

## 3. Results

**Figure 4:**
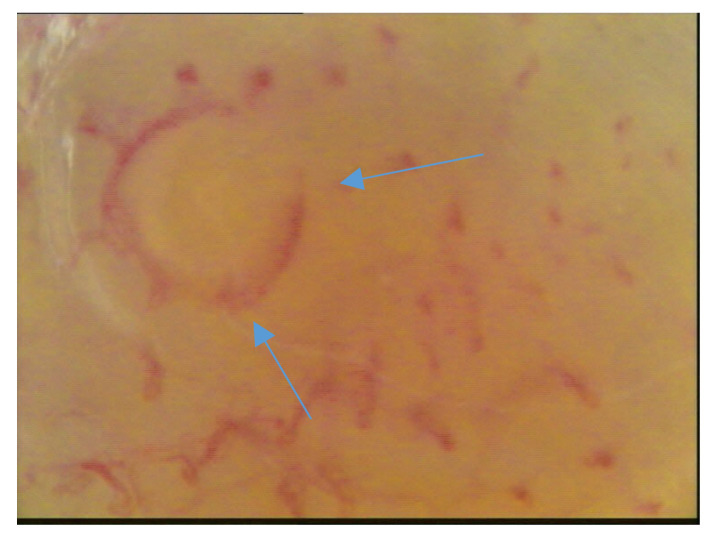
Capillaroscopic “sun” pattern in neuropathic diabetic foot (NDF) patients.

**Figure 5:**
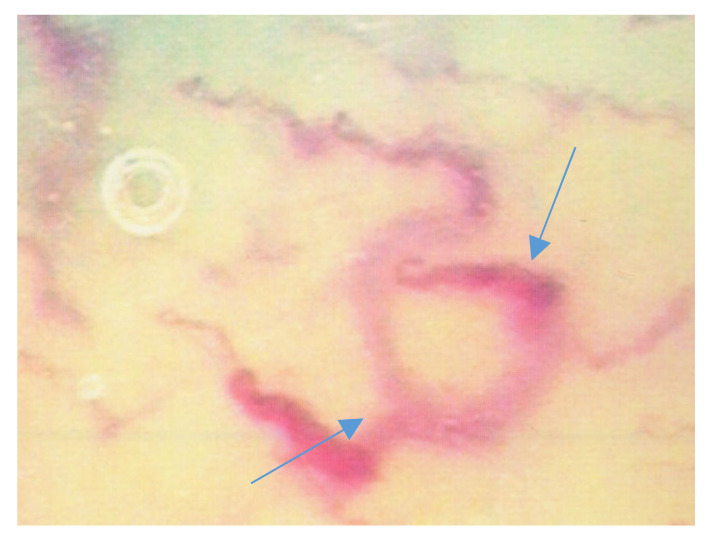
Capillaroscopic “sun” pattern in NDF patients.

The mean age for each group was: 66.43 ± 10.64 (range 44–78) for HS, 63.31 ± 12.01 (rang 28–72) for WFU, 63 ± 9.3 (range 43–79) for NDF and 75 ± 11.6 (range 52–97) for IDF. The M/F ratio was 8/7 in all the groups. The level of HbA1c in diabetic patients was 7.8% ± 0.6 (mean ± SD). In patients with neuropathic foot we observed: Semmes-Weinstein monofilament test < 4 ± 0.3 (mean ± SD); absence of patellar and Achilles reflexes in 12 patients; presence of deformity in 14 patients. In patients with ischemic diabetic foot we observed: wan color of the foot, low temperature and vascular distress observed by doppler examination, ABPI < 0.9 ± 0.1 (mean ± SD). The analysis of the morphological and densitometric characteristics of the capillaries revealed the following: a significant reduction in capillary density in neuropathic and ischemic patients compared to the control group of patients (both diabetic and healthy) (*p* < 0.05); a reduction in the average length of the capillaries and a significant increase in tortuosity (*p* < 0.05). In the neuropathic patients, a recurrent capillaroscopic pattern that we defined as “sun” was found, with capillaries arranged radially around an avascular area (Figure 4 and Figure 5) (Table 2 and Table 3).

## 4. Discussion

As there are no studies in literature that have shown “in vivo” alterations in the microcirculation of patients with diabetic foot, our study aimed to identify the micro-vascular changes in patients with ischemic and neuropathic diabetic foot.

The data emerging from this preliminary study suggest a potential diagnostic role of oral capillaroscopy in the early and subclinical identification of microangiopathic damage in patients with diabetic foot.

Diabetic foot represents a social and economic problem, becoming one of the leading causes of disability in the world [16].

Diabetic foot disease represents a serious complication in patients with diabetes, including lower extremity infection, ulcer formation and/or deep tissue damage, caused by a combination of neuropathy and vascular disease.

It can be classified in neuropathic foot, ischemic foot and neuro-ischemic foot. However, finding a purely ischemic foot, i.e., not associated with signs of neuropathy, is rare [9,10,17,18,19,20].

Any damage that occurs in the foot of a diabetic patient can cause complications that can lead to the amputation of the entire limb, especially if the treatment is delayed or ineffective [1,10].

Diabetic foot ulcers are the result of a complex pathophysiological mechanism that involves intrinsic factors, such as neuropathy, peripheral vasculopathy and extrinsic factors such as trauma, which is often unnoticed due to the loss of proprioceptive sensitivity, secondary to neuropathy [17,18,19].

The depression of neutrophils and of the bactericidal antioxidant systems determine the onset of infections that can involve the deep tissues and extend to the whole limb, ultimately resulting in loss of the limb through amputation [2,12].

The role of micro-angiopathy in the genesis of ulcers is still controversial today [19,21].

This is probably related to the difficulty of studying the microcirculation in vivo.

A recent study has shown profound histopathological alterations at the microcirculation level in patients with diabetic foot that could justify the genesis of lesions [21]. However, it is generally accepted that there is a close correlation between microcirculation anomalies and neuropathy in diabetic patients, demonstrated by the reduction in nerve conduction, vibratory sensitivity, and in the alteration of the sudomotor function [2,5,6,7,19,20,21].

The most frequent vascular hemodynamic alterations in these patients reside in arteriovenous shunt openings and in alterations in the vasomotor response [20].

The correct identification of vascular damage at an early stage could reveal a group of patients at risk, therefore deserving a close follow-up in order to prevent the genesis of ulcers.

In the event that microangiopathy is generalized, an important contribution can be ascribed to the study of peripheral microcirculation and therefore also to the study of oral microcirculation [22,23,24].

In recent years, the great potential of oral videocapillaroscopy as a preferential means of investigation for the “in vivo” study of microcirculation has been demonstrated [13].

The oral mucosa, in fact, has many advantages compared to other sites (periungual fold, conjunctival mucosa, dermis) [13,22,23,24,25].

Our study shows a correlation between this feared complication of diabetic disease and alterations in oral microcirculation.

A significant reduction in capillary density in neuropathic and ischemic patients compared to the control group of patients has been observed; a reduction in the average length of the capillaries and a significant increase in tortuosity are other oral microvascular alterations showed.

Further studies could be useful to monitor the oral microcirculation in diabetic patients at risk of “diabetic foot” in order to implement a therapeutic/pharmacological management capable of preventing this complication.

The difficulty in managing the disease when this complication arises is known.

It is due to the loss of the perfusion capacity of peripheral microcirculation.

Despite the obvious alterations, further studies are needed to correlate peripheral damage to the vascular patterns of the oral mucosa that we have identified.

The authors noted that the diabetic foot complication correlates with an alteration in oral peripheral microcirculation. However, the severity of the disease does not determine a further modification of the oral capillaroscopic picture. Therefore, the method can be used to assess the risk of the systemic disease developing the diabetic foot complication, but the onset of the clinical picture does not determine further significant changes in the oral microcirculatory pattern. Therefore, the monitoring of oral microcirculation, and thus its alteration in the diabetic patient, is a risk index that correlates with the systemic disease, which must then be monitored by the diabetic clinician with targeted tests and preventive therapies in order to avoid severe complications such as amputation.

The major limitations of the research are represented by the dimension of the enrolled subjects and this may be a limitation in inferencing the conclusion, but this is preliminary research.

Furthermore, the capillaroscopic investigations requires a serious learning curve for the operator.

“Quantitative Sensory Testing” may be required in future research design—e.g., thermal, pain, proprioception, vibration thresholds to evaluate how specific clinical manifestations of neuropathy. Neuropathic and vascular tests, bio tensiometer, Systoe, transcutaneous oxygen pressure, were used to confirm the diagnoses, but they were not directly obtained, and this is a limitation of this study. The study did not conduct any direct or indirect correlation analysis between density, length, tortuosity with the degree of neuropathy.

## 5. Conclusions

The study shows a significative correlation between the alterations of the oral microcirculation and the diabetic foot disease. Further research could highlight whether there is a prodromal role of oral microcirculatory changes and the risk of diabetic foot onset.

## Figures and Tables

**Table 1:** Baseline characteristics of the subjects enrolled in the study.

Characteristics of the Subjects	HS	WFU	NDF	IDF
Mean age	66.43 ± 10.64	63.31 ± 12.01	63 ± 9.3	75 ± 11.6
Age range	44–78	28–72	43–79	52–97
Male/Female ratio	8–7	8–7	8–7	8–7
Mean diabetes duration	0	21 ± 6.9 years	22.6 ± 12.0 years	23.7 ± 11.6 years

Data are mean ± SD. Level of significance between HS (Healthy Subjects) to WFU (Without Ulcerative Foot lesions), NDF (Neuropathic Diabetic Foot), IDF (Ischemic Diabetic Foot): *p* > 0.05 (ANOVA test) Not Significant (NS).

**Table 2:** Capillary density (% differences with HS).

Variables (Mean ± SD)	Lower Lip	Retro-Commissural Buccal Mucosa	Masticatory Mucosa
***HS*** n/mm^3^	19.04 ± 3.16	17.43 ± 2.34	26.74 ± 3.45
***WFU*** n/mm^3^	12.98 ± 3.1 (−31.83%) (S)	11.63 ± 3.7 (−33.3%) (S)	19.74 ± 2.4 (−26.2%) (S)
***NDF*** n/mm^3^	7.32 ± 2.1 (−61.6%) (S)	6.21 ± 3.4 (−64.4%) (S)	13.56 ± 2.6 (−49.3%) (S)
***IDF*** n/mm^3^	4.32 ± 3.2 (−77.3%) (S)	3.2 ± 1.7 (−81.6%) (S)	6.5 ± 2.1 (−75.7%) (S)

Level of significance between HS to WFU, NDF, IDF (ANOVA test and Bonferroni *t* test): *p* < 0.05 Significant (S).

**Table 3:** Capillary length (% differences with HS).

Variables (Mean ± SD)	Lower Lip	Retro-Commissural Buccal Mucosa
***HS*** μm	0.203 ± 0.023	0.245 ± 0.05
***WFU*** μm	0.383 ± 0.013 (+88.7%) (S)	0.366 ± 0.04 (+49.4%) (S)
***NDF*** μm	0.190 ± 0.04 (−6.4%) (S)	0.205 ± 0.02 (−16.3%) (S)
***IDF*** μm	0.178 ± 0.02 (−12.3%) (S)	0.232 ± 0.05 (−5.3%) (NS)

Level of significance between HS to WFU, NDF, IDF (ANOVA test and Bonferroni *t* test): *p* < 0.05 Significant (S); *p* > 0.05 Not Significant (NS).
